# Effect of the silencing of the *Ehcp112* gene on the *in vitro* virulence of *Entamoeba histolytica*

**DOI:** 10.1186/1756-3305-6-248

**Published:** 2013-08-28

**Authors:** Ramón Ocádiz-Ruiz, Wendy Fonseca, Máximo B Martínez, Ramón Ocádiz-Quintanar, Esther Orozco, Mario A Rodríguez

**Affiliations:** 1 Departamento de Infectómica y Patogénesis Molecular, CINVESTAV-IPN, A.P. 14-740, México, D.F., México; 2 Universidad Autónoma de la Ciudad de México, San Lorenzo No. 290. Colonia Del Valle. Delegación Benito Juárez. C.P 03100, México, D.F., México

**Keywords:** *Entamoeba histolytica*, Cysteine proteinases, EhCP112, siRNA-mediated silencing

## Abstract

**Background:**

*Entamoeba histolytica* is an intestinal protozoan parasite that causes amoebiasis in humans, affecting up to 50 million people worldwide each year and causing 40,000 to 100,000 deaths annually. EhCP112 is a cysteine proteinase of *E. histolytica* able to disrupt cell monolayers and digest extracellular matrix proteins, it is secreted by trophozoites and it can be active in a wide range of temperature and pH. These characteristics have encouraged the use of EhCP112 in the design and production of possible vaccines against amoebiasis, obtaining promising results. Nevertheless, we have no conclusive information about the role of EhCP112 in the *E. histolytica* pathogenesis.

**Methods:**

A set of three specific siRNA sequences were used to silence the *Ehcp112* gene via the soaking system. Silencing was evaluated by Western blot using an antibody against the EhCP112 recombinant protein. Finally, we analyzed the protease activity, the phagocytosis rate and the ability to destroy MDCK cells of the EhCP112-silenced trophozoites.

**Results:**

The highest silencing effect on EhCP112 was detected at 16 h of treatment; time enough to perform the *in vitro* virulence assays, which showed that EhCP112 silencing produces a significant reduction in cytolysis and phagocytosis of target cells, indicating the participation of this proteinase in these events.

**Conclusions:**

EhCP112 is involved in the *in vitro* virulence of *E. histolytica*.

## Background

*Entamoeba histolytica* is the intestinal protozoan parasite that causes amoebic colitis and amoebic liver abscesses in humans [1], affecting up to 50 million people worldwide each year and causing 40,000 to 100,000 deaths annually [2]. The cytopathogenic mechanism of this parasite consists of three main steps: adherence, cytolysis, and phagocytosis [3] and several molecules, including cysteine proteinases (CPs), participate in these events [4].

CPs have been identified as crucial virulence factors of protozoa parasites because they are associated to cytoadherence, haemolysis, cytotoxicity, degradation of extracellular matrix components, evasion of the immune response and nutrient acquisition [5-10]. The characterization of CP genes and their products have confirmed their relevance in invasion and colonization of host tissues [11-20]. As in other parasites, CPs play an important role in the pathogenicity of *E. histolytica*[21]. The genome of this parasite contains around fifty genes encoding CPs, although only few of them are expressed at significant levels in trophozoites grown in culture media [22,23]. Those CPs not expressed *in vitro* are thought to play a role in infection of the human host, invasion and destruction of host tissue and completion of the parasite life cycle [21].

EhCPADH is a protein complex of *E. histolytica* formed by the EhCP112 cysteine proteinase and the EhADH112 adhesin [24]. This complex is located in cytoplasmic vesicles and plasma membrane of trophozoites and is diminished in adherence- and virulence-deficient mutants [25]. EhCP112 contains a putative transmembranal domain and a RGD sequence for interaction with integrins [24]. The recombinant EhCP112 protein (rEhCP112) disrupts cell monolayers, digests extracellular matrix proteins and is active in a wide range of temperature and pH [26,27]. The results obtained with the recombinant protein suggest that EhCP112 could be involved in the pathogenic mechanism of this parasite; however, the relevance of EhCP112 in the virulence of *E. histolytica* has not been proved directly.

Here, we found that RNAi-mediated downregulation of EhCP112 in *E. histolytica* trophozoites leads to a reduction of the *in vitro* virulence of the parasite.

## Methods

### *Entamoeba histolytica* cultures

Trophozoites of clone A [28], strain HM1: IMSS, were axenically cultured in TYI-S-33 medium and harvested during the logarithmic growth phase as previously described [29].

### Expression and purification of the recombinant EhCP112 protein

An *Ehcp112* gene fragment (1278 bp), which encodes a polypeptide containing the pro-peptide and the mature enzyme, cloned in the expression vector pTrcHIs C [26] was used to transform bacteria *E. coli*, strain BL21 (pLys E). These bacteria were incubated with 1 mM of Isopropyl β-D-1-thiogalactopyranoside (IPTG) for 3 h at 37°C [30] to induce the production of the recombinant protein. Then, the EhCP112 recombinant protein (rEhCP112), containing a poly-histidine tag, was purified through Ni^2+^-affinity columns (Qiagen, USA) following the manufacturer’s protocol.

### Antibody generation

To obtain antibodies against rEhCP112, 100 μg of the recombinant protein was subcutaneously inoculated four times at intervals of 15 days on New Zealand rabbits. The first immunization was performed in the presence of complete Freund’s adjuvant (Sigma-Aldrich), and additional immunizations were carried out in the presence of incomplete Freund’s adjuvant (Sigma-Aldrich). The experimental protocol was approved by the institutional committee for animal care and provided all technical specifications for the production, care and use of laboratory animals (NOM-062-ZOO-1999).

### siRNA design

The complete *Ehcp112* mRNA sequence (GenBank: AF127375.2) was analyzed by the online program Target finder [31] to obtain potential small interference RNA (siRNA) sequences. Then, the multiple hypothetic sequences acquired were evaluated by nucleotide BLAST [32]*, and by the multiple sequence alignment ClustalW*[33], comparing all the sequences reported in the *Entamoeba* genome database Pathema [34], including all *E. histolytica* CPs. A set of three specific siRNA oligonucleotide sequences for *Ehcp112* were chosen (Table 1) and they were sent to their synthesis (Ambion). As a negative control, an additional set of scrambled sense and antisense primers with a non-related sequence (NRS) were synthesized.

**Table 1:** siRNA sequences

	
**siRNA 1**	Target sequence: 5′-AATCAAAAGACTCAAAGCATT-3′
	Sense strand: 5′-UCAAAAGACUCAAAGCAUUUU-3′
	Antisense strand: 5′-AAUGCUUUGAGUCUUUUGAUU-3′
**siRNA 2**	Target sequence: 5′-AATTGCTGTAAAATCCTTTTC-3′
	Sense strand: 5′-UUGCUGUAAAAUCCUUUUCUU-3′
	Antisense strand: 5′-GAAAAGGAUUUUACAGCAAUU-3′
**siRNA 3**	Target sequence: 5′-AAATATTTGTAGTTCATGTGT-3′
	Sense strand: 5′-AUAUUUGUAGUUCAUGUGUUU-3′
	Antisense strand: 5′-ACACAUGAACUACAAAUAUUU-3′

### siRNA preparation

All primers were diluted to 1 μg/μl in TE (10 mM Tris–HCl, 1 mM EDTA) and then, respective sense and antisense oligonucleotides were hybridized using the 1X DNA Annealing Solution (Ambion) incubating at 90°C for 3 min and then at 37°C for 1 h.

### Soaking of *E. histolytica* trophozoites with siRNA

Trophozoites (1 × 10^6^) freshly collected from 90% confluent cultures were inoculated in 25 ml culture plastic flasks (Corning) containing TYI-S-33 medium and incubated at 37°C during 24 h. Then the annealed siRNAs at 25, 50 and 100 μg/ml were added to the cultures and incubated at 37°C for 16, 24, and 36 h. To verify the siRNA entry to trophozoites by this soaking method, 75 pmol of a fluorescein-labeled negative control siRNA (Ambion) were added to 1 × 10^6^ trophozoites during 2, 5 and 16 h and internalization of the labeled-siRNA was analyzed by confocal microscopy. To determine the effect of the EhCP112 silencing on growth, the number of trophozoites and its viability was analyzed by trypan blue staining each 12 h after soaking with siRNA.

### Western blot assays

Total extracts from *E. histolytica* were separated by 10% SDS–PAGE and transferred to nitrocellulose membranes. Then, membranes were incubated with antibodies against rEhCP112 (1:10,000), followed by incubation with a mouse anti-rabbit IgG secondary antibody conjugated to horseradish peroxidase (Invitrogen) (1:10,000). Finally, the antibody detection was developed by incubation with 3,3′-diaminobenzidine tetrahydrochloride (Sigma-Aldrich) and H_2_O_2_. As internal control, the same membranes were revealed with anti-actin antibodies (1: 20,000). The bands detected by the anti-EhCP112 antibodies were analyzed by densitometry and data were normalized with those obtained from bands recognized by the anti-actin antibodies.

### Protease activity

To analyze the protease activity, trophozoites were washed twice with phosphate-buffered saline (PBS) and suspended at concentration of 10^7^/ml in PBS. The cellular pellets were lysed by five freeze-thaw cycles in liquid N_2_ and 10 μl of a 1:1000 dilution were mixed with 10 μl of 2× sample buffer without 2-mercaptoethanol and electrophoresed in 10% SDS-PAGE copolymerized with 0.1% gelatin. After electrophoresis, gels were incubated in 2.5% Triton ×-100 for 1 h at room temperature. CPs were activated by incubation in buffer activation (Tris–HCl 0.1 M pH 6.8, CaCl_2_ 10 mM and 0.02% 2-mercaptoethanol) for 12–16 h at 37°C. Gels were stained with 0.25% Coomassie blue R-250 and clear bands were indicative of proteolytic activity.

### Cytotoxic and cytophatic assays

MDCK (Madin-Darby canine kidney) monolayers (1 × 10^5^ cells) grown in 24-well plates (Costar) were incubated with 1 × 10^5^ trophozoites (cytopathic assays) or with total extracts from 1 × 10^6^ trophozoites (cytotoxic assays) in the presence of 0.02% of 2-mercaptoethanol during 2 h at 37°C. After this time, trophozoites or their extracts were eliminated and the remaining monolayers were washed three times with PBS, fixed with 2.5% glutaraldehyde, and cellular destruction was evaluated as previously described [35]. Briefly, cells were stained with 1% methylene blue, washed extensively and the dye captured by the rest of the monolayers was extracted with 0.1 N HCl and read in a spectrophotometer (Bechman coulter DU800) at 660 nm. Results were reported as the means ± standard deviation of three independent experiments by duplicate.

### Erythrophagocytosis

Erythrophagocytosis was measured using human red blood cells (hRBCs) as previously described [36], with some modifications. Fresh hRBCs (O Rh+) from healthy donors were washed three times and resuspended in serum-free amoeba culture medium. Then, trophozoites were incubated with hRBCs at a ratio of 1:100 [28] at 37°C with slight agitation for 5 and 10 min, the standard gap of time in the study of phagocytosis in *E. histolytica*[28]. Then, the non-ingested erythrocytes were lysed by incubation with distilled water for 10 min at room temperature and trophozoites were washed three times with PBS. Finally, samples were centrifuged at 360 × g during 5 min and pellets of amoebae with internalized hRBCs were resuspended in 1 ml of concentrated formic acid (J.T. Baker). Finally, the absorbance at 405 nm of the samples was measured against a blank of formic acid using a spectrophotometer (Beckman coulter DU800). Data were normalized using the formula *A*_405_ of sample/ *A*_405_ of control trophozoites (NRS-treated) at 10 min of phagocytosis. Results were reported as the means ± standard deviation of the percentage of normalized values of three independent experiments by duplicate.

## Results and discussion

### Generation of antibodies against EhCP112

EhCPADH is a protein complex formed by a CP (EhCP112) and an adhesin (EhADH112) [24]. This complex is located in cytoplasmic vesicles and plasma membrane of trophozoites [24,25]. EhCP112 and EhADH112 have been independently characterized [24,26,27,37]. In particular, the study and characterization of EhCP112 suggest that this protein could play an important role in the virulence of *E. histolytica*[24,26,27,38], but there is not a conclusive study about the role of EhCP112 in the *E. histolytica* pathogenesis.

The RNAi technology has proved to be a strong tool in molecular biology to study the functional analysis of cellular genes [39,40] and also for the development of novel therapeutic drugs to treat various incurable diseases [41,42]. Indeed, this technology has been useful in the study of *E. histolytica*[43-47]. Consequently, to confirm that EhCP112 is involved in the *E. histolytica* pathogenesis, we analyzed the *in vitro* virulence of EhCP112-silenced trophozoites.

**Figure 1:**
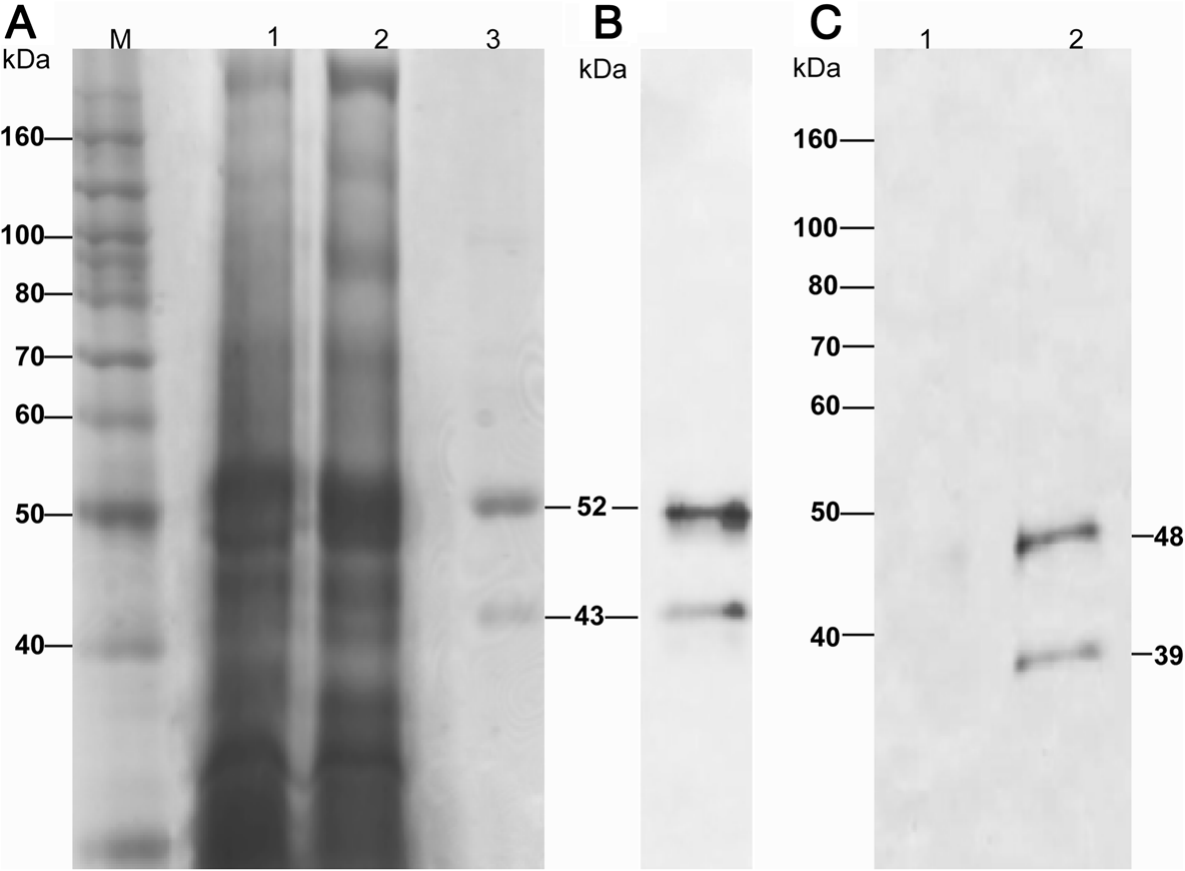
**Expression and purification of rEhCP112 and antibodies generation. (A)** Expression and purification of rEhCP112. An *Ehcp112* gene fragment (encoding the pro-peptide and the mature enzyme) cloned in the pTrcHIs C expression vector was expressed in *E. coli*. Then, the rEhCP112 was purified through Ni^2+^-affinity columns and analyzed by SDS-PAGE. Lane M, molecular weight markers; lane 1, uninduced bacteria extracts; lane 2, bacteria extracts induced by IPTG; lane 3, purified proteins corresponding to the pro-peptide and mature enzyme (52 and 43 kDa). **(B)** Antibodies generation. The rEhCP112 was inoculated in rabbits. Then, serum was tested by Western blot assays using the rEhCP112 in a SDS-PAGE. **(C)** Western blot on *E. histolytica* extracts. To analyze the specificity of the antibody against rEhCP112 we performed Western blot assays on total extracts of *E. histolytica* trophozoites; Lane 1, control using the preimmune serum; lane 2, antibody against rEhCP112.

To analyze the EhCP112 silencing, first we obtained specific antibodies against the recombinant EhCP112 (rEhCP112) [26]. Thus, the pTrcHIs-EhCP112 construction, encoding the pro-peptide and the mature enzyme [26], was expressed in bacteria *E. coli*, strain BL21 (pLys E). In SDS-PAGE of extracts of induced bacteria we observed a slight enrichment of a 52-kDa protein (Figure 1A), the expected molecular weight of rEhCP112 containing the poly-histidine tag encoded by the vector. Then, through Ni^2+^-NTA affinity columns we purified two polypeptides of 52 and 43 kDa (Figure 1A). These polypeptides correlate with the expected molecular weight of the pro-enzyme (52 kDa) and the mature enzyme (43 kDa) variants of EhCP112 containing the poly-histidine tag, suggesting that rEhCP112 is an active enzyme [26].

In order to generate antibodies against EhCP112, New Zealand rabbits were subcutaneously inoculated with 100 μg of the purified rEhCP112. Then, the antibodies obtained were tested by Western blot. In these assays, two bands were recognized in both the recombinant protein and trophozoite extracts (Figure 1B, C). These bands correspond to the expected molecular weight of the pro-enzyme and the mature enzyme. No bands were detected by the preimmune serum in trophozoite extracts (Figure 1C), suggesting that antibodies are specific against EhCP112.

### siRNA design

Once we had specific antibodies against EhCP112, we designed the siRNA sequences using the complete mRNA sequence of this CP (GenBank: AF127375.2) as target. The online program Target finder [31] predicted 70 hypothetic target sequences. To determine the specificity of these potential target sites, we used the BLAST tool of the *Entamoeba* genome database Pathema [34] and the NCBI BLAST tool [32]. We eliminated from consideration any target sequence with more than 10 contiguous base pairs of homology to other coding sequences in *E. histolytica.*

A set of three specific EhCP112 siRNA oligonucleotide sequences were chosen (Table 1). These sequences are located towards the 5′-end (siRNA 1), in the middle of the mRNA sequence (siRNA 2), and towards the 3′end (siRNA 3), they did not show significant homology to other mRNAs encoding *E. histolytica* genes, included other CPs. As a negative control, an additional set of non-related sense and antisense primers were used.

### Internalization of siRNA

**Figure 2:**
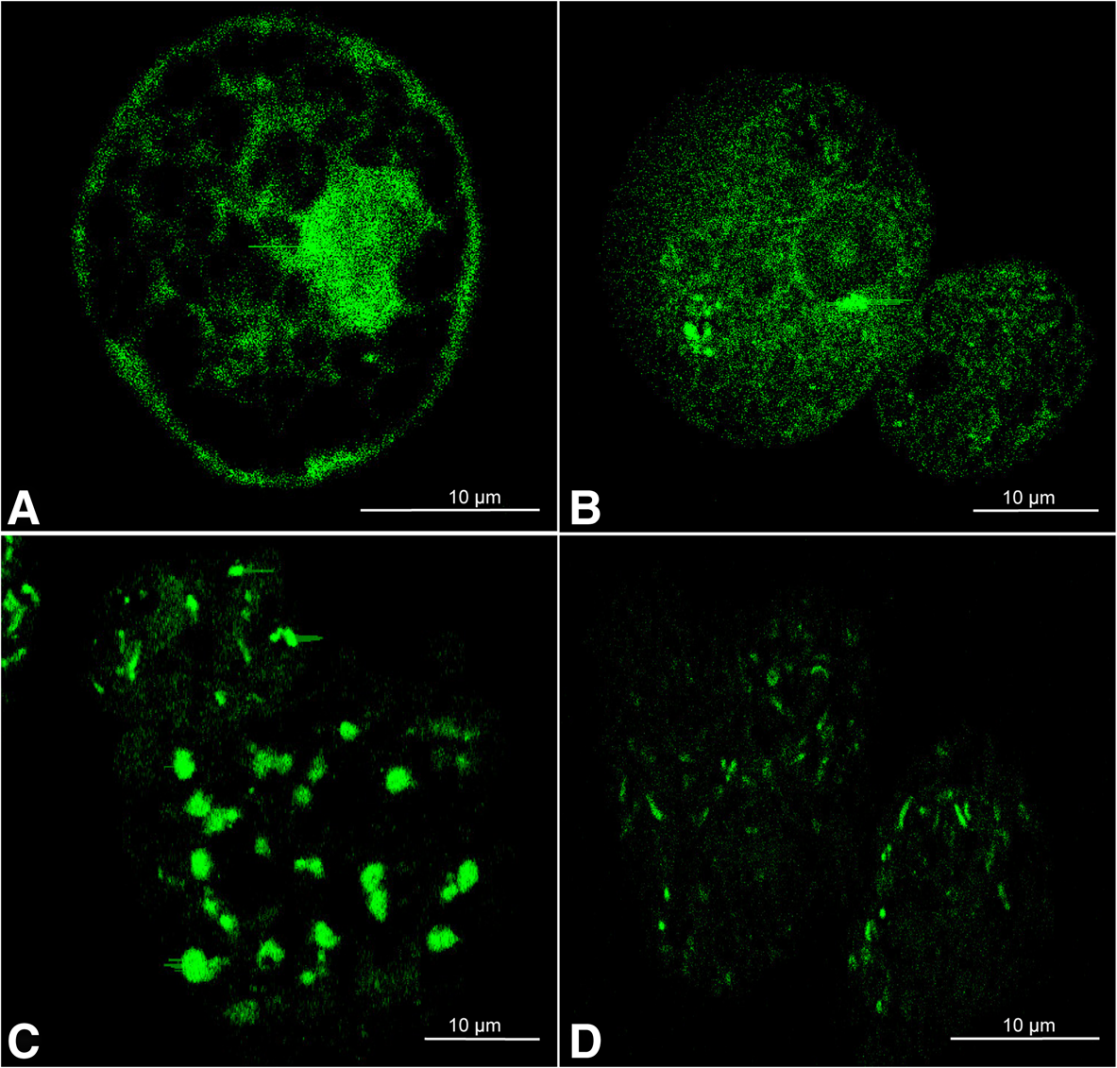
**Analysis of internalization of a fluorescein-labeled siRNA.** Trophozoites were incubated at several times with a fluorescein-labeled siRNA via soaking method. The entry to trophozoites was verified by confocal microscopy. **(A)** 5 minutes incubation; **(B)** 2 hours incubation; **(C)** 5 hours incubation; **(D)** 16 hours incubation.

Before performing the EhCP112-silencing, the entry of siRNA into the trophozoites by the soaking method was verified by confocal microscopy using a fluorescent-labeled siRNA. In these experiments we observed that since 5 min of incubation, the siRNA was bound to the plasma membrane of the trophozoites, and a weak punctuated label was detected inside the trophozoites (Figure 2A). At 2 h of incubation the labeled-siRNA was detected dispersed in the interior of the trophozoites (Figure 2B); and at longer times (5 and 16 h), siRNA was observed as fluorescent spots inside the trophozoites (Figure 2C, D), but these spots were smaller after 16 h of incubation (Figure 2D). These results showed that siRNAs are efficiently internalized into the trophozoites by the soaking method.

### EhCP112 silencing

**Figure 3:**
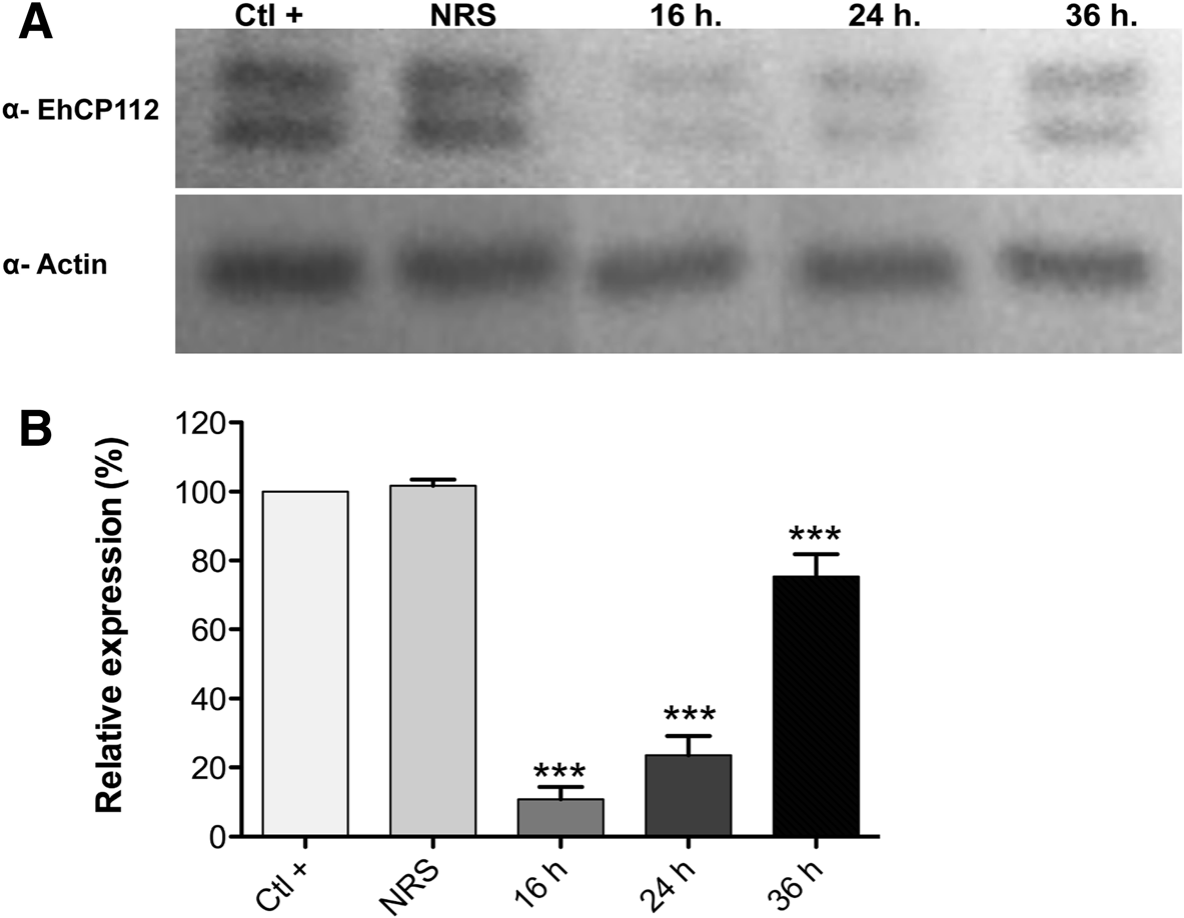
**EhCP112 silencing.** Trophozoites were incubated at different times with 50 μg/ml of the siRNA 1 via the soaking method and the silencing of EhCP112 was analyzed by Western blot assays. **(A)** Western blot. Total extracts obtained from non-treated trophozoites (Ctl+), trophozoites incubated with a double-strand RNA with non-related sequence (NRS) and trophozoites incubated with the siRNA 1 at different times (16, 24 and 36 h) were analyzed by Western blot using the antibodies against rEhCP112. As internal control, membranes were also incubated with an anti-actin antibody. **(B)** Relative expression of EhCP112. The bands recognized by antibodies were analyzed by densitometry. The EhCP112 expression was normalized to actin expression and the value obtained in non-treated trophozoites was taken as 100% of relative expression. Asterisks: *P* < 0.001.

In order to evaluate the silencing of EhCP112, we analyzed its expression by Western blot in trophozoites incubated with the three siRNAs sequences at three different concentrations (25, 50 and 100 μg/ml) for 16, 24 and 36 h. Each siRNA was tested separately and mixed. In these assays we observed a decreased expression of EhCP112 in trophozoites treated for 16 and 24 h with 50 μg/ml of the siRNA 1, corresponding to the 5′-end of the gen, whereas the EhCP112 expression was not affected by incubation with the siRNA control, containing a non-related sequence (NRS) (Figure 3A). As internal control we performed Western blot assays using an anti-actin antibody, which detected a 45-kDa band with almost the same intensity in all samples (Figure 3A). Densitometry of the bands detected, showed that the EhCP112 expression decreased in 89 and 76%, after 16 and 24 h of incubation, respectively (Figure 3B). However, after 36 h of incubation, the expression of EhCP112 decreased only 25% (Figure 3B), indicating that the silencing effect was reversible and dose dependent. Some authors have been reported that in eukaryotic cells, the siRNA in the RNAi process is degraded together with the mRNA, thus the process is not continue [41,42]. On the other hand, the EhCP112 silencing had no effect on the proliferation of the trophozoites (data not shown), suggesting that this CP is not needed for growth in culture medium.

The incubation of trophozoites with 100 μg/ml of siRNA 1 not increased the EhCP112 silencing (data not shown). In addition, siRNA 2 and siRNA 3 did not show a significant effect in the EhCP112 expression. Consequently, the incubation with the siRNA mixture did not increase the EhCP112 silencing caused by siRNA 1 (data not shown). Then, consecutive experiments were performed with trophozoites after 16 h of treatment with 50 μg/ml of siRNA 1, because in these conditions we observed the most efficient effect on the EhCP112 silencing.

### Effect of the EhCP112 silencing on the *E. histolytica in vitro* virulence

**Figure 4:**
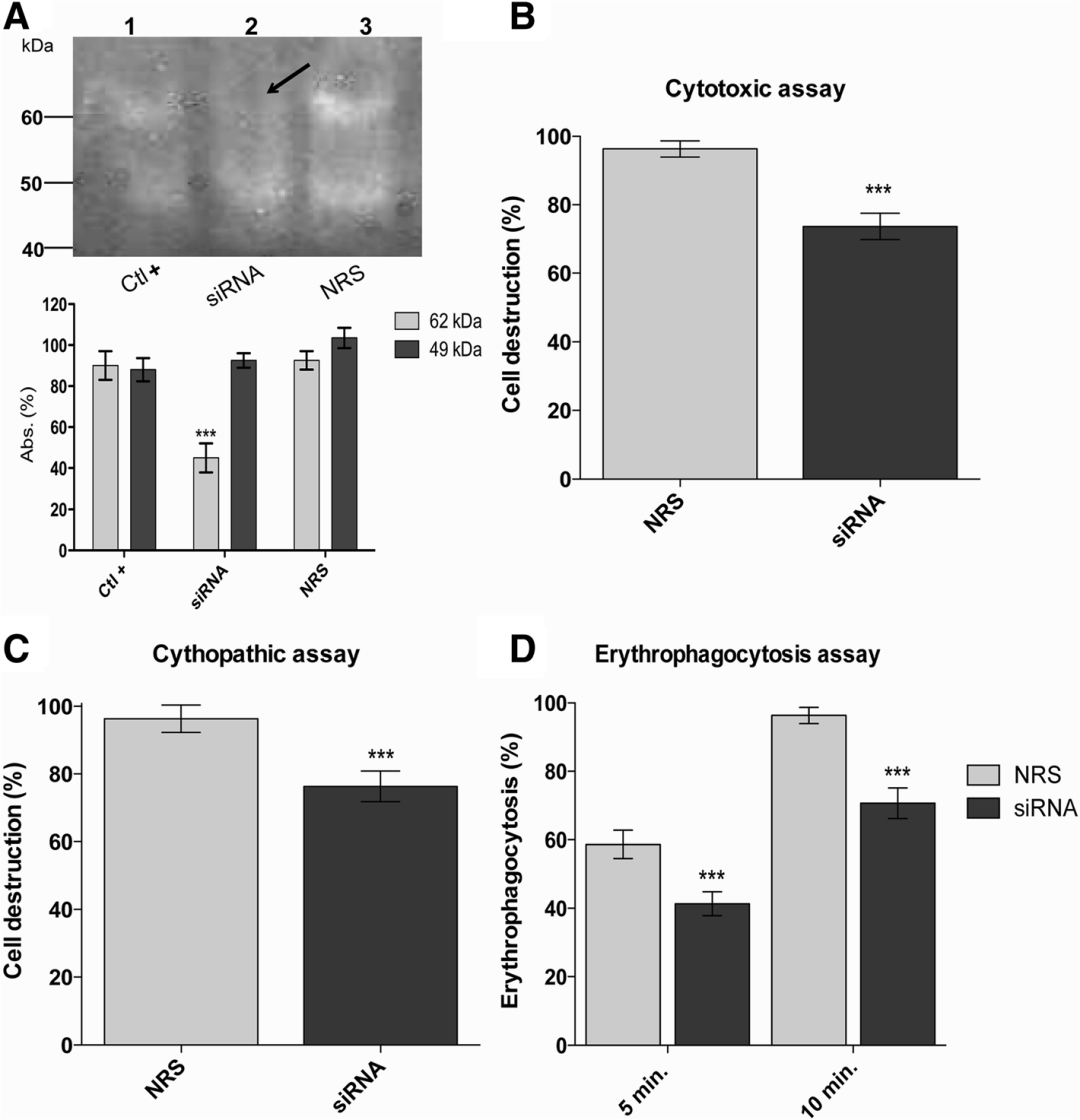
***In vitro *virulence of Ehcp112-silenced trophozoites. (A)** Protease activity. The protease activity of the trophozoites treated by 16 h with the siRNA 1 (EhCP112-silenced trophozoites) was analyzed in polyacrylamide-0.1% gelatin substrate gels. Lane 1, extracts from untreated trophozoites (Ctl+); lane 2, extracts from EhCP112-silenced trophozoites (SiRNA); lane 3, extracts from trophozoites treated with a non-related sequence (NRS). Arrow indicates the 62-kDa band with minor proteolytic activity in EhCP112-silenced trophozoites. Below is shown the densitometry of the proteinase activity of the 62- and 49-kDa bands. **(B)** Cytotoxic activity. Total extracts from EhCP112-silenced trophozoites (SiRNA) and NRS-treated trophozoites (NRS) were incubated with MDCK monolayers during 2 h. Then, the monolayer destruction was evaluated. **(C)** Cytopathic assays. EhCP112-silenced trophozoites (SiRNA) and NRS-treated trophozoites (NRS) were incubated with MDCK monolayers during 2 h. Then, the monolayer destruction was evaluated. **(D)** Erythrophagocytosis assays. EhCP112-silenced trophozoites (SiRNA) and NRS-treated trophozoites (NRS) were incubated with human red blood cells during 5 and 10 min. Then, the ingestion of the target cells was evaluated and value obtained with the NRS-treated trophozoites at 10 min of phagocytosis was arbitrary taken as 100% efficiency. Data were analyzed in three independent assays by duplicate and they are expressed as the means ± standard deviation. Differences were evaluated by t-student test. Asterisks: *P* < 0.001.

Once the EhCP112 silencing was achieved, we determined its effect on the *in vitro* virulence of *E. histolytica*. First, the protease activity of the silenced trophozoites was analyzed in polyacrylamide-0.1% gelatin substrate gels. The EhCP112-silenced trophozoites showed a 62-kDa band with smaller proteinase activity to that of trophozoites treated with the non-related sequence (NRS) and untreated trophozoites (Figure 4A). Densitometry of that band revealed a 55% minor activity in EhCP112-silenced trophozoites with respect to controls, whereas the 49-kDa band showed similar activity in all conditions (Figure 4A). The 62-kDa band could correspond to the active EhCP112 enzyme; its higher molecular weight is probably due to the native electrophoretic conditions. Nevertheless, these assays suggested that EhCP112 silencing has a significant effect only in one band with protease activity in substrate gels.

In order to analyze the cytotoxic activity of total extracts from EhCP112-silenced trophozoites, total amoeba extracts were incubated with MDCK monolayers during 2 h and then the monolayer destruction was evaluated. Results showed that silenced trophozoites presented a significantly reduction in the cytotoxic activity (25%) in relation to the NRS-treated trophozoites (Figure 4B), suggesting that EhCP112 has a relevant role in cytolysis.

In these assays we evaluated the monolayer cell destruction by total trophozoite extracts, where all the amoeba cysteine proteinases have been released. Nevertheless, there are few cysteine proteinases, including EhCP112, reported to be secreted that could be involved in cytolysis [26,48]. To analyze the role of EhCP112 in cell destruction by trophozoites, we performed cytopathic assays using EhCP112-silenced trophozoites on MDCK monolayers. These experiments showed a significant decrease of 25% in cell destruction by the EhCP112-silenced trophozoites in comparison with the cell destruction performed by the NRS-treated trophozoites (Figure 4C), eliciting the EhCP112 activity in cell destruction and tissue invasion.

We also carried out erytrophagocytosis assays, because phagocytosis is an event involved in the pathogenic mechanism of *E. histolytica*[28]. Thus, we evaluated the ingestion of human red blood cells (RBC) during 5 and 10 min by the EhCP112-silenced and NRS-treated trophozoites. For these assays, the phagocytic value obtained from NRS-treated trophozoites at 10 min of phagocytosis was arbitrary taken as 100% efficiency. At 5 min of incubation, the difference between the EhCP112-silenced and the NRS-treated trophozoites was 19%; silenced trophozoites showed a phagocytosis efficiency of 44%, whereas NRS-treated trophozoites displayed an efficiency of 63% (Figure 4D). At 10 min of incubation, the difference between these populations was 34%; silenced trophozoites showed 66% of phagocytosis efficiency (Figure 4D). These results suggest that EhCP112 could be involved in the phagocytic activity of *E. histolytica*.

Data obtained in this study support the hypothesis that EhCP112 is involved in the pathogenicity of *E. histolytica*. However, despite that expression of EhCP112 decreased around 89% after incubation with siRNA, cell cytotoxicity, cytopathic effect and erythrocyte phagocytosis between siRNA and NRS treated trophozoites showed minor, but significant, differences (25 to 34%). These results suggest that other mechanisms might also contribute in these processes.

On the other hand, it has been reported that the expression of the homologue of EhCP112 in *E. invadens*, a related parasite of reptilians, is increased in the cyst stage [49], suggesting that this CP could also participate in development of the life cycle in *Entamoeba sp*. The EhCP112 silencing could be useful to test this possibility; unfortunately the *in vitro* encystation of *E. histolytica* is not still available.

## Conclusions

In this study we achieved the silencing of EhCP112 by siRNA. The highest silencing effect was detected at 16 h of treatment; time enough to perform the *in vitro* virulence assays, which showed that EhCP112 silencing produces a significant reduction in cytolysis and phagocytosis, indicating the participation of EhCP112 in these events. These results and those obtained with the EhCP112 recombinant protein [26] support the use of EhCP112, in combination with other virulence factors of *E. histolytica*, as a therapeutic target or a vaccine candidate.

## Abbreviations

BLAST: Basic Local Alignment Search Tool; CPs: Cysteine proteinases; EhCP112: Cysteine proteinase of *E. histolytica* which is part of the EhCPADH protein complex; hRBCs: Human red blood cells; IPTG: Isopropyl β-D-1-thiogalactopyranoside; MDCK: Madin-Darby canine kidney cell line; NRS: Non-related sequence; PBS: Phosphate buffered saline; rEhCP112: EhCP112 recombinant protein; siRNA: Small interference RNA.

## Competing interests

The authors declare that they have no competing interests.

## Authors’ contributions

ROR and WF conceived and carried out experiments, analyzed data and drafted the manuscript; MBM, ROQ and EO participated in the design of the study, analyzed data and helped to draft the manuscript; MAR conceived and designed the study, analyzed data and drafted the manuscript. All authors read and approved the final manuscript.
